# Supplementary material for: Identification of photoperiod-induced specific miRNAs in the adrenal glands of Sunite sheep (Ovis aries)
Source: Front Vet Sci. 2022 Jul 22;9:888207. doi: 10.3389/fvets.2022.888207 (PMC9354845; doi:10.3389/fvets.2022.888207)
Supplement: Supplementary file 1 [file Data_Sheet_1.ZIP › Supplementary file/miRDeep2 file.zip!/miRDeep2 file/Novel_Structure/SP42A2/Novel_314.pdf]

Provisional ID	:	NC_019484.2_42176
Score total	:	2.4
Score for star read(s)	:	3.9
Score for read counts	:	-3.3
Score for mfe	:	0.3
Score for randfold	:	1.6
Score for cons. seed	:	
Total read count	:	5
Mature read count	:	4
Loop read count	:	0
Star read count	:	1

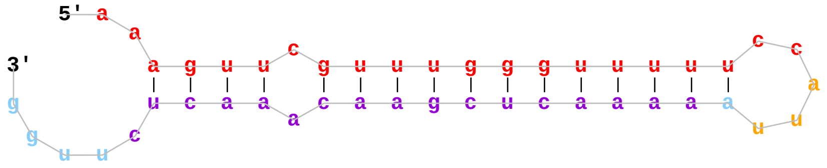

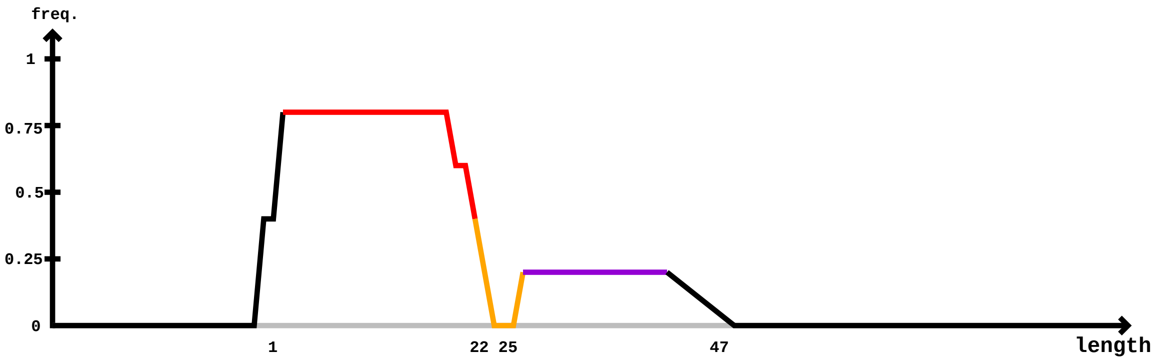

Mature Star

```
5'- uguuaaauaauggauuggcugcaaaguucguuuggguuuuuccauuaaaaacucgaacaaacucuugggccaacccaauauaagaguuacagccuuuaucugcagugugu  -3'   obs
    uguuaaauaauggauuggcugcaaaguucguuuggguuuuuccauuaaaaacucgaacaaacucuugggccaacccaauauaagaguuacagccuuuaucugcagugugu         exp
    ......(((.(((.((((((.(((((((.((((((((((((.....)))))))))))).)))).))))))))).))).)))......(((.((........)).)))...   reads    mm    sample
    ....................gcaaaguucguuuggguuuu......................................................................   1        0     seq
    ....................gcaaaguucguuuggguuuuuc....................................................................   1        0     seq
    ......................Uaaguucguuuggguuuuucc...................................................................   2        1     seq
    ...............................................aaaacAcgaacaaacuc..............................................   1        1     seq
```
